# Laponite^®^/Ethyl Lauroyl Arginate Complex–Functionalized Silk Peptide/Chitosan Edible Coating for Banana Preservation

**DOI:** 10.3390/foods15213821

**Published:** 2026-10-26

**Authors:** Congcong Zhao, Dongxia Hu, Nina Mo, Guanghui Wang, Hongying Lin, Minmin Tang, Yayuan Zhang, Xiaozhi Tang, Ru Zhao, Tingli Liu, Zhenjiong Wang

**Affiliations:** 1 College of Food Science and Engineering, Nanjing University of Finance and Economics, Collaborative Innovation Center for Modern Grain Circulation and Safety, Nanjing 210023, China; 2 School of Food Science, Nanjing Xiaozhuang University, Nanjing 211171, China; 3 Key Laboratory of Food Contact Materials Safety, State Administration for Market Regulation, Jiangsu Product Quality Testing & Inspection Institute, Nanjing 210007, China; 4 Institute of Agro–Products Processing Science and Technology, Guangxi Academy of Agricultural Science, Nanning 530007, China

**Keywords:** chitosan, silk peptide, Laponite^®^, ethyl lauroyl arginate, biopolymer film, banana preservation

## Abstract

Laponite^®^ (LRD) was modified with ethyl lauroyl arginate (LAE) to form complexes, which were subsequently incorporated into solutions of silk peptide (SP) and chitosan (CS) to prepare SP–CS biopolymer composite films by the casting method. Compared with LRD, the LRD/LAE complex demonstrated superior dispersibility within the SP–CS film, thereby improving the compatibility between SP and CS. The mechanical, thermal and antimicrobial properties of the SP–CS film increased while the water vapor permeability (WVP) decreased with the incorporation of the LRD/LAE complex. Remarkably, the SP–CS film containing 2% LRD and 1% LAE complex (SP–CS–C) exhibited the lowest WVP (0.35 g·mm·m^−2^·h^−1^·kPa^−1^) resulted in the highest tensile strength (27.99 MPa) and elongation at break (81.62%). When an edible bananas coating was applied, the weight loss of the SP–CS–C film decreased to 13.92%, and the hardness (176.94 g), titratable acidity (0.15%), and total soluble solids (19.20%) improved. The microbial counts decreased to 3.80 log CFU/g for the colonies and 3.30 log CFU/g for the molds on day 7, whereas they decreased to 5.78 and 4.65 log CFU/g, respectively, in the control group. These results show that the SP–CS–C film extends the banana shelf life during storage at 25 °C and approximately 50% RH for 7 days.

## 1. Introduction

In recent years, petroleum–derived plastics have dominated food packaging applications because of their low cost and versatility. However, their nonrenewable nature and resistance to biodegradation have raised significant environmental concerns. Consequently, the development of sustainable alternatives has become an urgent priority [1,2]. Chitosan (CS), a polysaccharide synthesized through the deacetylation of chitin, has emerged as a promising candidate because of its inherent biodegradability, biocompatibility, nontoxicity, and film–forming capabilities [3,4]. However, the commercial use of pure chitosan films in food packaging is usually hampered by their low mechanical strength and high sensitivity to moisture [5]. To overcome these shortcomings, the combination of chitosan with other biopolymers is an effective strategy for improving the overall performance of composite films. This approach not only alleviates the shortcomings of individual constituents but also imparts new functionalities to the composite material [6,7]. Silk peptide (SP) is a low–molecular–weight hydrolysate of silk fibroin that is highly water soluble and easy to process, making it an appropriate reinforcing agent [8]. Chitosan and silk fibroin derivatives have received significant attention as composite films because they can be utilized to improve the mechanical strength and barrier properties [9]. Although the study by Kweon et al. supports this viewpoint [10], balancing the mechanical strength and barrier performance is still challenging for practical packaging applications.

Another useful pathway for strengthening biopolymer matrices is the incorporation of nanofillers. Laponite^®^ (LRD) is a hectorite–type nanoparticle synthetic with a disk geometry (the thickness of the nanoparticle is approximately 1 nm, and the diameter of the nanoparticle is approximately 25 nm) [11]. Its empirical equation Na^+^_0.7_[(Si_8_Mg_5.5_Li_0.3_)O_20_(OH)_4_]^−^_0.7_ indicates isomorphic replacement of magnesium by lithium, which leaves the disk faces with a net negative charge [12]. Laponite^®^ is a good synthetic nanofiller that is biocompatible and can be incorporated into sustainable food packaging to provide added value for UV barrier properties to overcome the drawbacks of traditional biopolymer packaging [13]. Although LRD has potential, it is prone to aggregation because of high interlayer interactions and is strongly hydrophilic, thus resulting in low interfacial compatibility with organic polymer matrices [14]. Thus, it is necessary to organically modify it to increase the spacing between layers, reduce aggregation, and improve hydrophobicity [15]. Although traditional modifiers most frequently use quaternary ammonium salts or other artificial surfactants, there is an increasing need for green, food–grade alternatives [16]. LAE is an antimicrobial cationic surfactant from natural sources (L–arginine, lauric acid, and ethanol) that is approved by the FDA as a safe antimicrobial food preservative and has a broad spectrum of activity [17,18]. LAE features a hydrophilic cationic head group and a hydrophobic tail, making it an ideal candidate for modifying negatively charged clays. Compared with other commonly used modifiers, such as quaternary ammonium salts or metal nanoparticles, LAE has several advantages: it is a food–grade compound approved for direct food contact by regulatory agencies (e.g., the FDA and EFSA); it exhibits broad–spectrum antimicrobial activity against Gram–positive and Gram–negative bacteria, yeasts, and molds; and it is biodegradable with low toxicity, making it particularly suitable for use as an edible coating. In our previous work, we successfully utilized LAE in starch/PBAT films to extend the shelf life of strawberries [19]. Furthermore, preliminary findings suggest that LAE can intercalate into LRD galleries, thereby increasing layer spacing and imparting antibacterial properties.

Although SP–CS films have been investigated for food preservation, the combined effects of LRD/LAE complexes on their structure, functional properties, and preservation performance remain insufficiently understood. Therefore, in this study, we hypothesized that LAE modification could improve the dispersion of LRD and its compatibility with the SP–CS matrix, thereby enhancing the performance of the resulting composite films. To our knowledge, this is the first study to incorporate LRD/LAE complexes into an SP–CS matrix and evaluate their potential for banana preservation. The effects of different LAE concentrations on the structure, morphology, and functional properties of LRD were first examined, after which the LRD/LAE complexes were incorporated into SP–CS films to assess their effects on film morphology and mechanical, barrier, thermal, and antimicrobial properties. Finally, the optimized composite film was applied as an edible coating to evaluate its effectiveness in maintaining banana quality and extending its shelf life. By connecting the structural modification of LRD with the properties of the composite film and its preservation performance, this study addresses an important gap in the development and practical application of SP–CS–based edible packaging materials.

## 2. Materials and Methods

### 2.1. Reagents and Materials

LAE was obtained from Beijing HWRK Chemical Co., Ltd. (Beijing, China). LRD was purchased from BYK Additives (Shanghai) Co., Ltd. (Shanghai, China). Silk peptide (Mw = 1.5 × 10^4^ g/mol) was acquired from China Tiansi Biotech Co., Ltd. (Huzhou, China). Low–molecular–weight chitosan (Mw 5 × 10^4^ g·mol^−1^, min 90% deacetylation degree) was purchased from Jinan Haidebei Biological Co., Ltd. (Jinan, China). Acetic acid (98 wt% purity) was supplied by SCR (Shanghai, China).

### 2.2. Preparation of the LRD/LAE Complex

To prepare the LRD/LAE complex, preliminary screening experiments were carried out to define the working concentration range of LAE. Concentrations below 0.5% *w*/*v* led to inadequate LAE loading and weak LRD exfoliation, whereas LAE concentrations above 1.5% *w*/*v* easily caused phase separation and excessive brittleness of the SP–CS films. Thus, LAE concentrations of 0.5, 1.0 and 1.5% *w*/*v* were adopted. Briefly, LRD (2% *w*/*v*) was dispersed in deionized water and magnetically stirred at 25 °C and 800 rpm until fully dispersed. The suspension was sonicated for 5 min using an ultrasonic generator (SCIENTZ-1500F, Ningbo Scientz Biotechnology Co., Ltd., Ningbo, China) to obtain a homogeneous LRD suspension. LAE was then added to the LRD suspension at the target LRD:LAE ratio, followed by continuous magnetic stirring at 800 rpm for 12 h to form a uniform LRD/LAE suspension. After freeze-drying at −80 °C, the resulting LRD/LAE complexes were named 2–0.5C, 2–C and 2–1.5C and stored at room temperature prior to use. Here, 2–0.5C, 2–C and 2–1.5C correspond to 2% LRD complexed with 0.5%, 1.0% and 1.5% LAE, respectively.

### 2.3. Preparation of SP–CS Films Containing LRD/LAE Complexes

**Figure 1:**
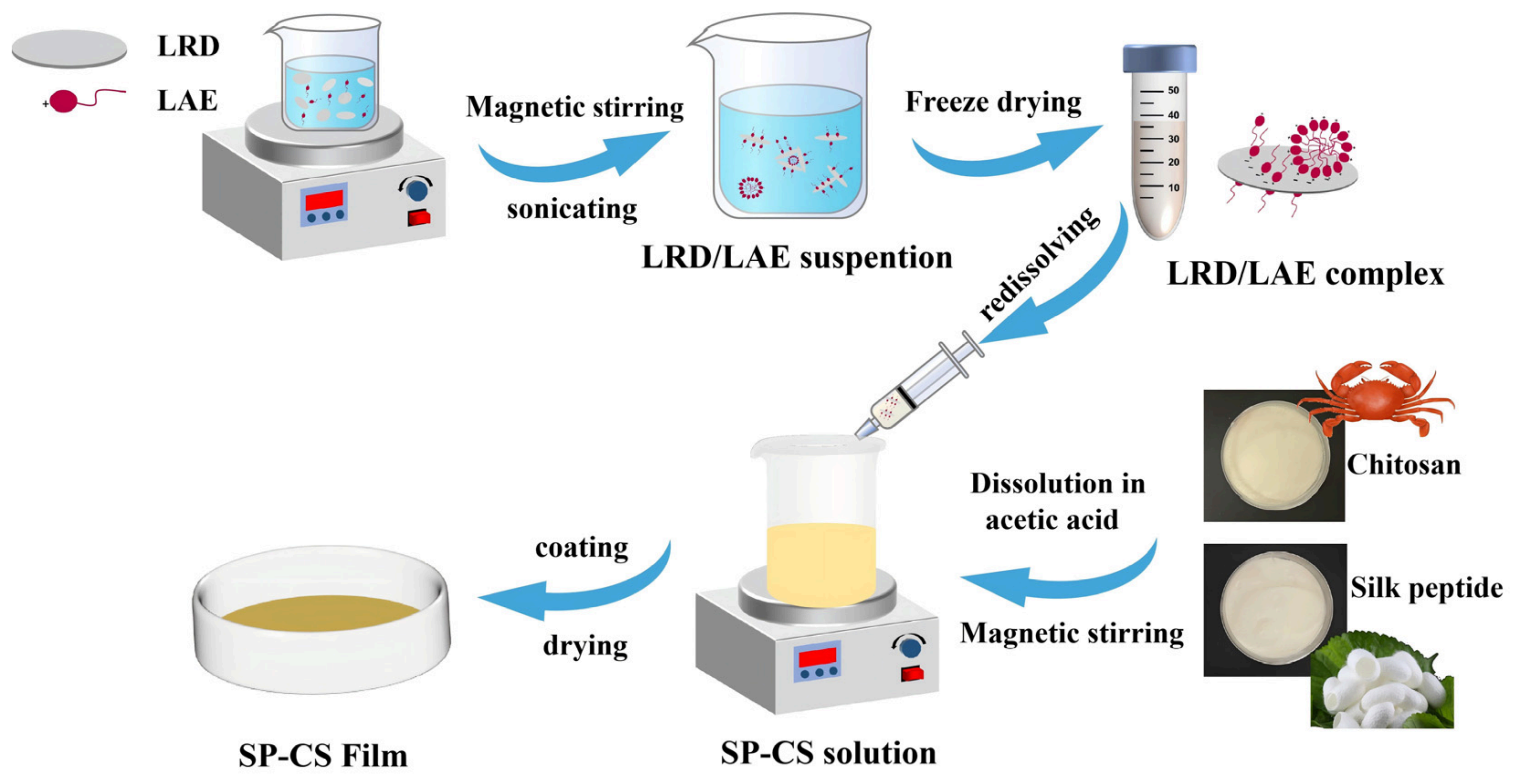
The preparation process of the SP–CS films containing the LRD/LAE complex.

Stock solutions of chitosan (CS) and silk peptide (SP) were prepared separately by dissolving 2 g of each powder into 100 mL of 2% (*w*/*v*) acetic acid solution. The mixtures were stirred magnetically at room temperature for 4 h to ensure complete dissolution. To prepare the film–forming matrix, the SP and CS solutions were blended at a weight ratio of 6:4 (SP:CS). The LRD/LAE complexes (denoted as LRD, 2–0.5C, 2–C and 2–1.5C, respectively) were subsequently added directly to the film–forming solution at a loading of 5 wt% relative to the total weight of the solid biopolymers (SP and CS). The resulting mixtures were stirred continuously at 60 °C for 2 h to obtain homogenous film–forming solutions. Finally, the solutions were cast into a 13 cm × 13 cm mold and dried in an oven at 40 °C for 12 h. Prior to characterization, the peeled films were conditioned in a humidity chamber at 25 ± 0.5 °C and 50 ± 2% relative humidity (RH) for 48 h. The control film was designated as SP–CS. The nanocomposite films were labeled SP–CS–LRD, SP–CS–0.5C, SP–CS–C, and SP–CS–1.5C, corresponding to the addition of unmodified LRD and LRD modified with increasing concentrations of LAE (2–0.5C, 2–C, and 2–1.5C, respectively). The preparation schematic is illustrated in Figure 1, and the detailed formulations are listed in Table 1.

### 2.4. Characterization

The films were cut into thin strips (20 × 10 mm) and fractured in liquid nitrogen. Both the surface morphology and the cross–sectional morphology of the films were examined through a scanning electron microscope (TM3000, Hitachi, Tokyo, Japan) at 15 kV. FTIR spectra of the films were recorded using a Fourier transform infrared spectrometer (Spectrum Two, PerkinElmer, Waltham, MA, USA) equipped with an attenuated total reflectance (ATR) accessory. The scanning range was 4000–400 cm, with 32 scans and a resolution of 4 cm^−1^. For secondary structure analysis, the amide I region (1600–1700 cm^−1^) was baseline corrected and deconvoluted with Gaussian functions using Nicolet Omnic and PeakFit V4.12, with assignments based on the literature [20]; fits with R^2^ > 0.99 were accepted. The crystallinity of the films was assessed by an X–ray diffractometer (D2 Phaser, Bruker, Karlsruhe, Germany). Scans were conducted at a tube voltage of 40 kV and a tube current of 40 mA over a 2θ diffraction angle range of 2° to 60° at a scanning speed of 1°/min using CuKα radiation. The thermal stability of the films was studied using a thermogravimetric analyzer (TGA550, TA Instruments, New Castle, DE, USA). In a nitrogen atmosphere, the film (5 mg) was heated from 30 °C to 600 °C at a heating rate of 10 °C/min.

### 2.5. Film Properties

#### 2.5.1. Appearance, Thickness, Moisture Content (MC), Opacity and Transmission

The appearance of the films was documented using a digital camera. The thickness of the films was measured by a thickness gauge (CHY-C2A, Labthink, Jinan, China) according to our previously reported method [19]). The color of the films and banana peels was characterized by UV-vis absorbance spectra recorded with an ultraviolet–visible spectrophotometer (U-3900, Hitachi, Tokyo, Japan) over the range of 200–800 nm, as described below. The moisture content (MC) was determined following the weighing method of the AOAC (1995), with some modifications [21]. For this, the equilibrated film was cut into a 10 × 10 mm square (M0), dried in an oven at 105 °C for 12 h and then weighed as M1. The MC value was calculated using the following formula:

MC (%) = 100% × (M_0_ − M_1_)/M_0_ (1)

where M_0_ and M_1_ are the weights of the initial and dried films, respectively.

The absorbance of the films was also determined using an ultraviolet spectrophotometer (U−3900, Hitachi, Tokyo, Japan). The light transmittance of the films was used to represent the transparency. The opacity and transmission of the films were calculated according to the following formula:

Opacity = A_600_/d (2)

Transmission (%) = 10^−*A*^ × 100 (3)

where A is the absorbance of the film at 600 nm and d is the thickness (mm) of the film.

#### 2.5.2. Water Vapor Permeability (WVP)

The water vapor transmission rate (WVTR) and WVP of the films were determined according to ASTM E96–95 (ASTM, 2004) using a previously reported method [22]. The film was sealed onto a test cup containing 30 g of silica gel, weighed, and placed in a chamber at 25 °C and 50% RH. The cup was weighed again after 12 h. WVTR was calculated as WVTR = Δm/(A × t), and WVP was calculated as WVP = WVTR × d/ΔP, where Δm is the weight change (g), A is the exposed area (m^2^), t is the test time (h), d is the film thickness (mm), and ΔP is the difference in partial vapor pressure (kPa).

#### 2.5.3. Mechanical Properties

The tensile strength (TS) and elongation at break (EAB) of the films were determined using an electronic universal testing machine (UTM4104X, Shenzhen SUNS Technology Stock Co., Ltd., Shenzhen, China) in accordance with the ASTM D882–02 standard [23]. The films were cut into the same strips (20 × 40 mm) and positioned between grips with an initial grip distance of 30 mm and a cross–head speed of 10 mm/min.

### 2.6. Application to Banana

Banana is among the most consumed and economically important fruits worldwide, with high postharvest losses due to rapid ripening and senescence. Its sensitivity to mechanical damage, microbial spoilage, and enzymatic browning makes it an ideal model for testing the efficacy of edible coatings. The permeability of the peel to gases and its susceptibility to fungal infections provide a sensitive system for evaluating the barrier and antimicrobial properties of a coating. Bananas are characterized by their irregular shapes and soft peels. In contrast to wrapping with preformed films, which risks damaging the peel and often results in uneven coverage, the immersion coating method facilitates the formation of a uniform and conformal edible layer on the fruit surface. This approach ensures consistent contact between the coating material and the fruit while avoiding mechanical damage. Consequently, this application mode is widely adopted for the preservation of irregular fruits and vegetables [24,25] and aligns well with practical industrial requirements. For these experiments, fresh bananas (Cavendish) were purchased from the local fruit market in Nanjing, China. Bananas of uniform size, color, shape and maturity (yellowish green) were randomly divided into 3 groups with 30 fruits each, namely, the control group, the SP–CS group and the SP–CS–C group. The experimental unit was a single banana, with N = 6 independent fruits per treatment per sampling time. Different fruits were used at each sampling time (destructive sampling). At each time point (days 0, 1, 3, 5, and 7), six new fruits per treatment were randomly selected and evaluated. Bananas in the SP–CS group and the SP–CS–C group were immersed in the SP–CS and SP–CS–C film−forming solutions, respectively, for 30 s, ensuring complete surface coverage. The film−forming solution adhered to the banana surface and was dried to form a uniform coating, which was consistent with the practical application scenario of edible film. Afterward, all the bananas were naturally dried and stored at 25 °C with 50% RH. Quality changes were investigated by sampling at various storage durations (0, 1, 3, 5 and 7 days) to quantify the appearance, weight loss, firmness, total soluble solids content (TSS), titratable acid (TA) content and bacterial growth of the stored fruits.

A camera was used to record the changes in the appearance of the bananas. The weight loss (WL) of the bananas was determined by comparing their initial weight with their weight after storage [26]. The firmness of the bananas was measured using a texture analyzer equipped with an SMSP/6 probe (penetration distance 10 mm, speed 1 mm/s, ten replicates) on the basis of our group’s previously reported method [19]. For the TSS, 10 g of banana pulp was ground in a mortar and centrifuged at 12,000 rpm for 10 min; the supernatant was collected and measured directly using a refractometer. Each sample was measured three times, and the average value was recorded.

To determine the titratable acidity, 10 g of banana pulp was ground to 100 mL with distilled water [27]. A 20 mL aliquot of the supernatant was taken, mixed with 1% (*w*/*v*) phenolphthalein as an indicator, and titrated with 0.1 M NaOH until a light pink color appeared and persisted for 30 s. Titratable acidity was calculated using the formula TA (%) = (V × c × V_1_/V_2_ × m) × 0.067 × 100, where V is the volume of filtrate (mL), c is the concentration of NaOH (mol/L), V_1_ is the volume of NaOH consumed (mL), V_2_ is the volume of filtrate titrated (mL), m is the sample mass (g), and 0.067 is the malic acid conversion factor (g/mmol).

The total numbers of molds and colonies on banana peels were determined according to GB 4789.15−2016 (National food safety standard—Food microbiological examination—Enumeration of molds and yeasts) [28] and GB 4789.2−2022 (National food safety standard—Food microbiological examination—Determination of aerobic plate count), respectively [29]. Microbial enumeration was performed in triplicate (*n* = 3) per treatment at each sampling time, and the results are expressed as the mean ± SD (log CFU/g).

### 2.7. Statistical Analysis

All measurements were performed in triplicate, and the results are expressed as the mean ± standard deviation. For film characterization (thickness, moisture content, opacity, WVP and mechanical properties), the data were analyzed by one−way analysis of variance (ANOVA), followed by Duncan’s multiple range test at *p* < 0.05. For the banana storage experiment, a completely randomized design with three treatments (control, SP−CS and SP−CS−C) and five sampling times (0, 1, 3, 5 and 7 days) was used (N = 6 independent fruits per treatment per time, destructive sampling). Data were analyzed by two–way ANOVA with treatment and storage time as fixed factors, including the treatment × time interaction. Post hoc pairwise comparisons were performed using Duncan’s multiple range test at *p* < 0.05. Microbial counts were log10−transformed before analysis to satisfy normality assumptions. Normality (Shapiro–Wilk) and homogeneity of variance (Levene’s) were verified prior to ANOVA. Statistical analysis was performed using IBM SPSS 22.0, and figures were prepared using GraphPad Prism 8. The error bars represent the standard deviation; different letters denote statistically significant differences at *p* < 0.05.

## 3. Results and Discussion

### 3.1. FTIR Analysis

All the SP-CS films exhibited characteristic absorption bands associated with SP and CS, with their respective amino and amide bands overlapping and some characteristic band shifting. The displacements of the films were at 1411 cm^−1^ (pyrrolidine ring of SP), 1384 cm^−1^ (symmetric deformation mode of CH_3_ in CS) and 1157 cm^−1^ (asymmetric C−O−C stretching in CS glycosidic bonds). The bands at 1261 cm^−1^ (amide III of CS) and 698 cm^−1^ (amide V of SP) almost disappeared. These changes reflected hydrogen bond interactions between CS and SP, which have been extensively reported in previous literature [30,31,32]. In addition, the 1652 cm^−1^ (C=O stretching vibration) peak of CS merged with the 1620 cm^−1^ (C=O) peak of SP, and the 1599 cm^−1^ (−NH_2_ bending vibration) peak of CS gradually combined with the 1597 and 1520 cm^−1^ (N−H) peaks of SP, forming a broad peak. These changes may be attributed to the electrostatic interactions between the -NH_2_ groups of CS and the -COOH groups of SP [33,34]. The amide I region of SP is sensitive to its secondary structure. According to Hu et al. [35], the spectral region of 1600–1630 cm^−1^ is generally assigned to the β−sheet structure, with the 1630–1650 cm^−1^ band corresponding to random coils, the 1650–1660 cm^−1^ band corresponding to α–helices, the 1660–1680 cm^−1^ band corresponding to β−turns, and the 1680–1700 cm^−1^ band corresponding to antiparallel β−sheets. Therefore, the amide I bands of the different films were deconvoluted using PeakFit 4.12 to estimate the relative contributions of the corresponding secondary structural components.

**Figure 2:**
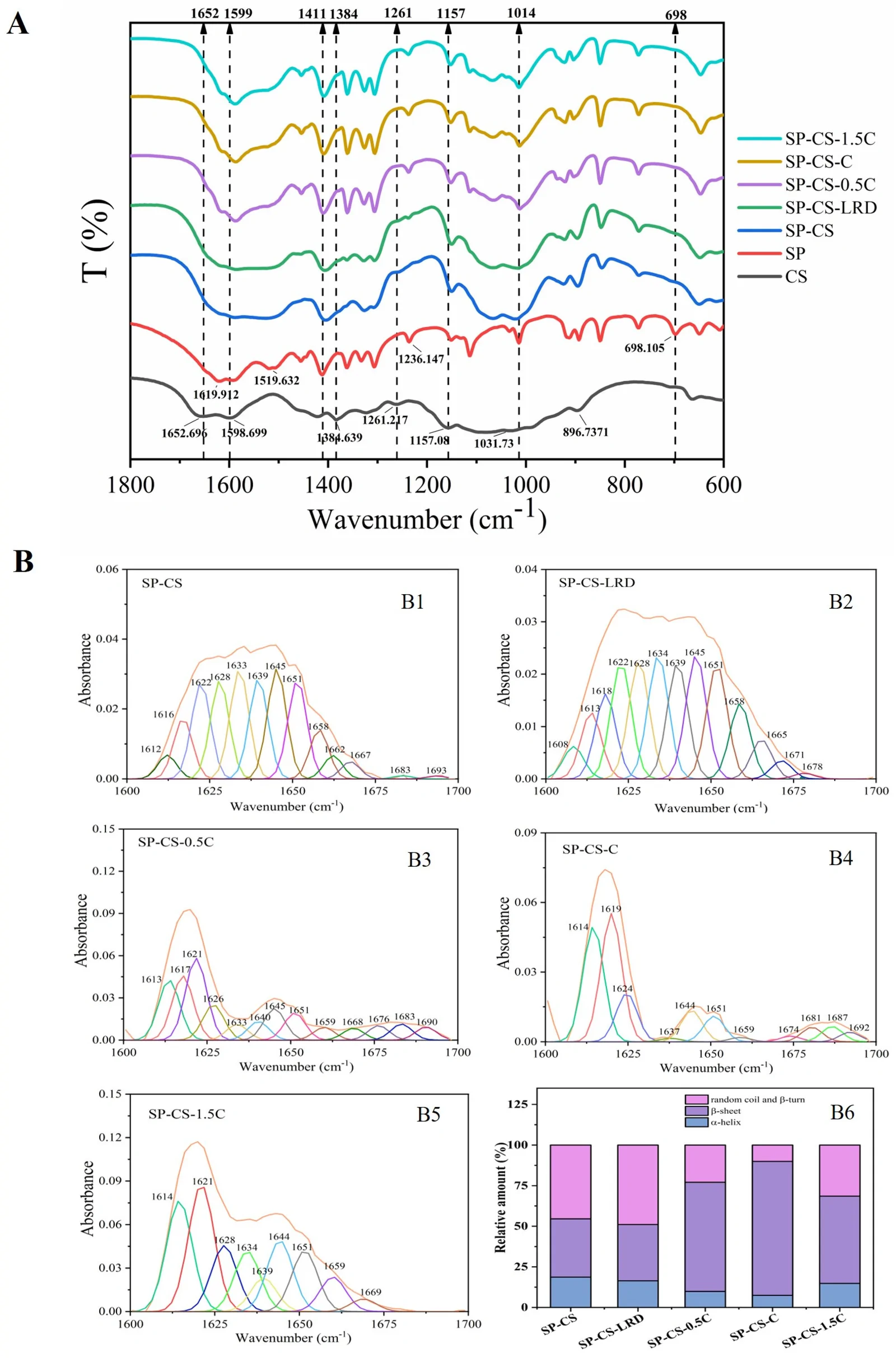
FTIR spectra (**A**) and curve–fitting results of the amide I band ((**B1**) SP−CS; (**B2**) SP−CS−LRD; (**B3**) SP−CS−0.5C; (**B4**) SP−CS−C; (**B5**) SP−CS−1.5C) and relative proportion of protein secondary structures (**B6**) of SP–CS films containing different LRD/LAE complexes.

As shown in Figure 2B, the fitted relative fractions of α−helix and β−sheet structures in the neat SP–CS film were 18.67% and 35.89%, respectively. The corresponding values for the SP−CS−LRD film were 16.42% and 34.62%, indicating that the addition of unmodified LRD was not associated with a marked change in the fitted secondary–structure composition of SP. This observation suggests that the incorporation of LRD did not markedly alter the secondary structure of SP, which may be related to the higher isoelectric point of CS than that of SP, potentially leading LRD to interact preferentially with the positively charged CS in the SP−CS matrix. Moreover, the inherent tendency of LRD to aggregate may hinder its interaction with SP and consequently affect its secondary structure. In contrast, the fitted β–sheet fractions of the SP−CS films containing different LRD/LAE complexes were significantly greater, with values of 67.24%, 82.49% and 53.71% for SP−CS−0.5C, SP−CS−C and SP−CS−1.5C, respectively. These changes in the amide I spectral profile suggest that incorporation of the LRD/LAE formulations altered the molecular organization of SP within the SP−CS matrix and was associated with an increase in β−sheet−related structural ordering. Spectral deconvolution revealed the highest β−sheet fraction in the SP−CS−C film, whereas a significant decrease in the β−sheet proportion occurred in the SP−CS−1.5C film. Such divergence in secondary structure composition among LRD/LAE−containing films implies that SP conformation is not controlled by a single factor. Instead, SP folding is modulated by a combination of intermolecular interactions generated during solvent evaporation and film drying, including LRD/LAE aggregation status, hydrogen−bond networks, electrostatic attraction and compactness of molecular packing. Overall, the FTIR results demonstrate that the incorporation of LRD/LAE complexes remodels intermolecular interactions and molecular arrangement within the SP−CS matrix. Variations observed in the amide I band point to the conformational rearrangement of SP toward a structure enriched in β−sheets. Considering the complexity of this system, such structural variations are more likely caused by the combined influence of LRD/LAE complexes.

### 3.2. X–Ray Diffraction

**Figure 3:**
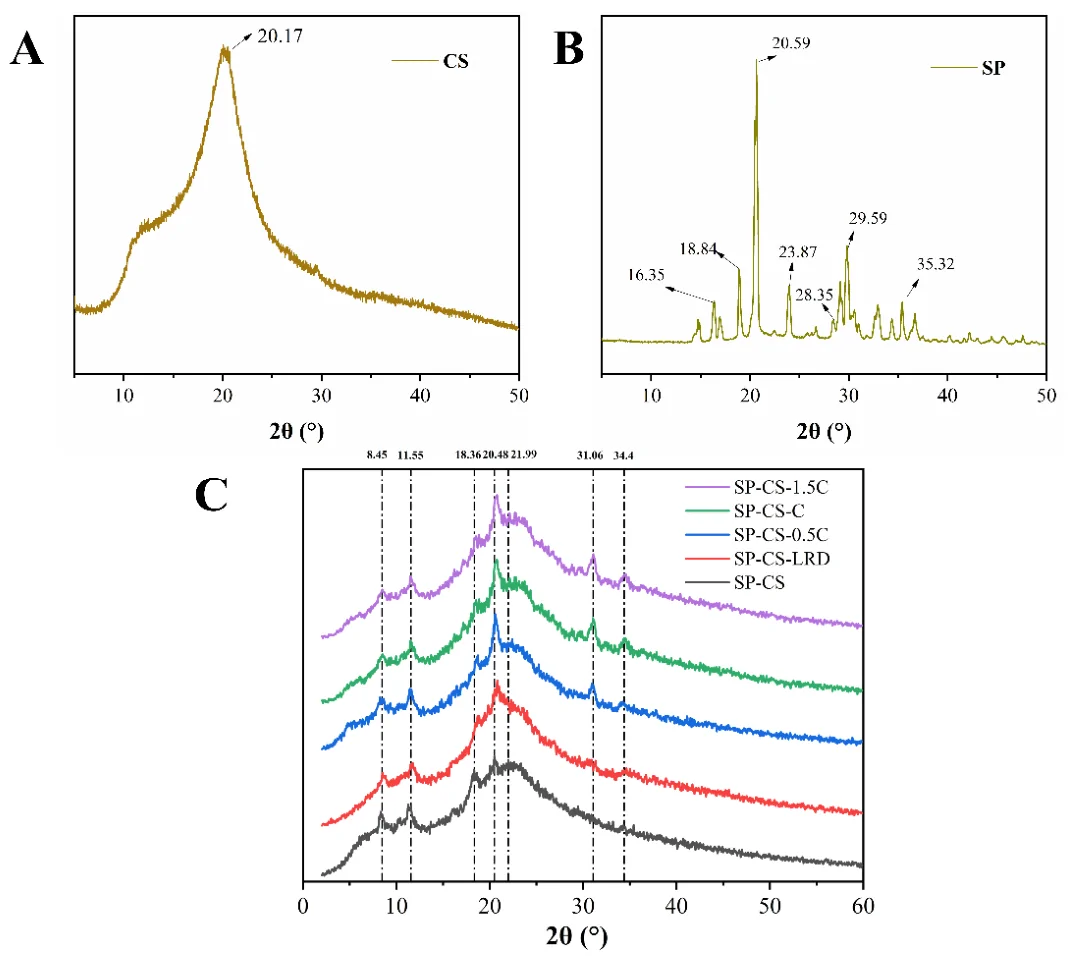
XRD patterns of the SP–CS films containing different LRD/LAE complexes ((**A**) CS; (**B**) SP; (**C**) SP–CS films containing different LRD/LAE complexes).

The crystal structures of the CS, SP and SP−CS films were analyzed by XRD. The SP−CS film displayed characteristic peaks associated with both CS and SP, with some peaks shifting (Figure 3C). The diffraction peak at 11.55° corresponded to the silk I conformation (α−helix) of SP [36]. The broad diffraction peak at approximately 20° resulted from the overlap of SP and CS. The peak at 21.99° was associated with CS, whereas the peaks at 18.36° and 20.48° represented the silk II conformation of SP (β−sheet) [31,34,37]. The new diffraction peak observed at 8.45° may be attributed to the formation of the “Form I” structure of CS following acetic acid treatment. The peak intensity of SP−CS−LRD was lower at 21.99°, indicating that LRD reduced the recrystallization of CS. Moreover, SP−CS−LRD exhibited a typical diffraction peak for LRD at 19.81°, indicating the aggregation of LRD in the SP and CS matrix. New peaks in all the SP−CS films with LRD/LAE complexes were observed at 31.06° and 34.4°, which corresponded to silk II (β−Sheet). In addition, the strength of the peak at 20.48° increased greatly, indicating that the LRD/LAE complex may facilitate the conversion of SP−CS into the Silk II conformation, potentially increasing crystallinity. These XRD findings were consistent with the FTIR results. These variations are presumably attributable to the conformational rearrangement of SP, along with improved structural ordering correlated with elevated β−sheet content upon incorporation of the LRD/LAE complex. Notably, however, this conformational transition was incomplete because the diffraction peaks that were related to the silk I conformation remained in the XRD patterns of the SP−CS films.

### 3.3. Scanning Electron Microscopy (SEM)

**Figure 4:**
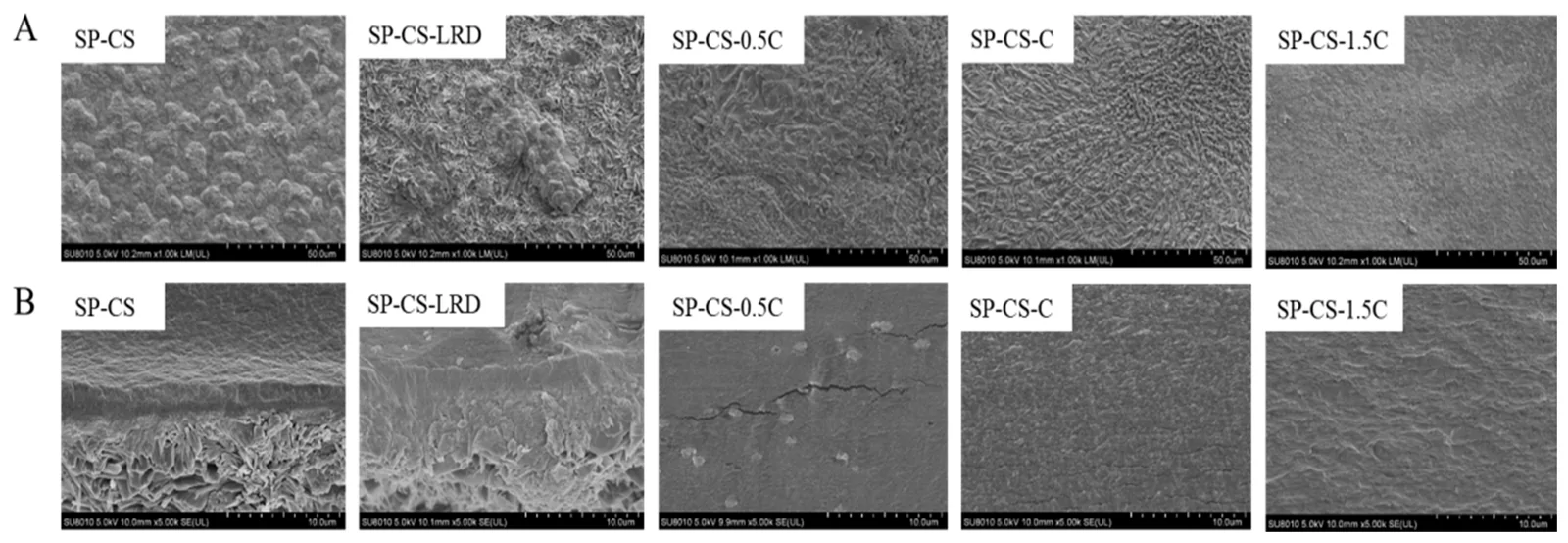
SEM images of the surface (**A**) and cross–section (**B**) of the SP–CS films containing different LRD/LAE complexes.

The surface and cross−sectional images of the SP–CS films with various LRD/LAE complexes are shown in Figure 4. The SP−CS film was found to be phase−separated because of the weak intermolecular forces between the SP and CS, making them incompatible [38]. The SP−CS−LRD film also underwent phase separation, and aggregates in the form of noticeable particles could be observed in the cross–section. This implies that LRD tends to collect and is difficult to spread evenly, which undermines film compatibility [39]. Nevertheless, the surface of the SP–CS film to which the LRD/LAE complex was attached was smooth and compact, with no apparent clustered LRD particles visible in the cross–section. This may be attributed to the fact that LRD altered by LAE may constitute exfoliated layers that were evenly distributed in the SP and CS matrix. These findings were consistent with the results of [21], who reported that the surface roughness of films with surfactants and nanoclay was lower than that of those with clay only [21]. Although these morphological observations suggest improved dispersion of LRD within the polymer matrix, they do not establish the biocompatibility or safety of the resulting films. The biocompatibility of LRD has been reported in the literature [40]. However, the biocompatibility and migration behavior of the present composite films were not evaluated in this study, and these aspects warrant further investigation in future work.

### 3.4. Thermogravimetric Analysis (TG)

**Figure 5:**
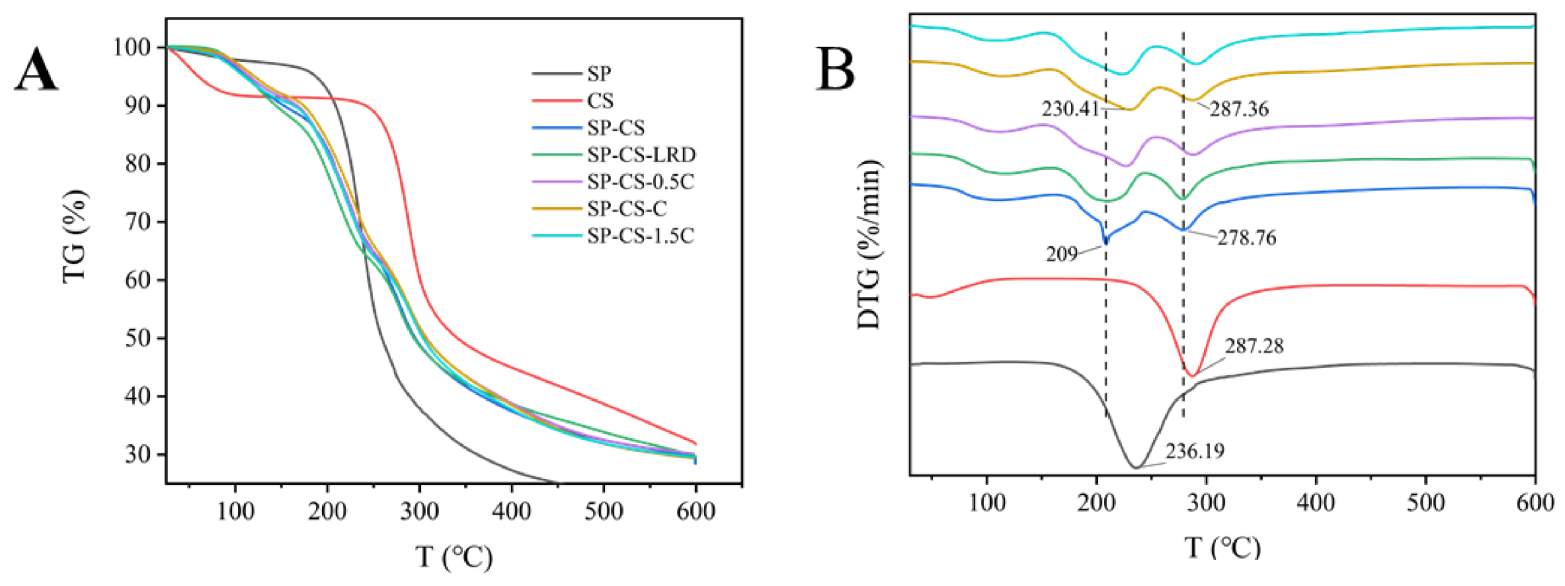
TG spectrum (**A**) and DTG spectrum (**B**) of SP–CS films containing different LRD/LAE complexes.

The TG and DTG curves of the SP−CS composite films are shown in Figure 5. The main loss of weight occurred in the 160–400 °C range, which corresponds to the thermal degradation of the organic constituents of the samples, namely, CS and SP (Figure 5A). As observed in the DTG curves (Figure 5B), the addition of LRD alone did not significantly affect the thermal stability of the film. In contrast, the addition of LRD/LAE complexes to the film markedly improved the thermal stability, with the SP−CS−C film performing optimally. In particular, the maximum weight loss rate temperature (Tm) of the SP component increased to 230.41 °C instead of 209.00 °C, whereas the maximum weight loss rate temperature (Tm) of the CS component increased to 287.36 °C instead of 278.76 °C. This increase can be primarily attributed to the LRD/LAE complex−induced conformational transition of SP from a silk I structure to a more thermally stable silk II structure [41]. Additionally, the weight loss rate of the SP−CS−C film decreased, suggesting that the optimal dispersion of the 2−1 LRD/LAE complex facilitated strong interactions with SP and CS, thereby resulting in the formation of a denser and more stable network structure. Furthermore, in the SP−CS films containing LRD/LAE complexes, the thermal decomposition peaks of the SP and CS components tended to merge. However, they failed to coalesce into a single broad peak, indicating that the LRD/LAE complexes only partially improved the compatibility between SP and CS.

### 3.5. Appearance, Thickness, Moisture Content (MC), Opacity and Transmission

**Figure 6:**
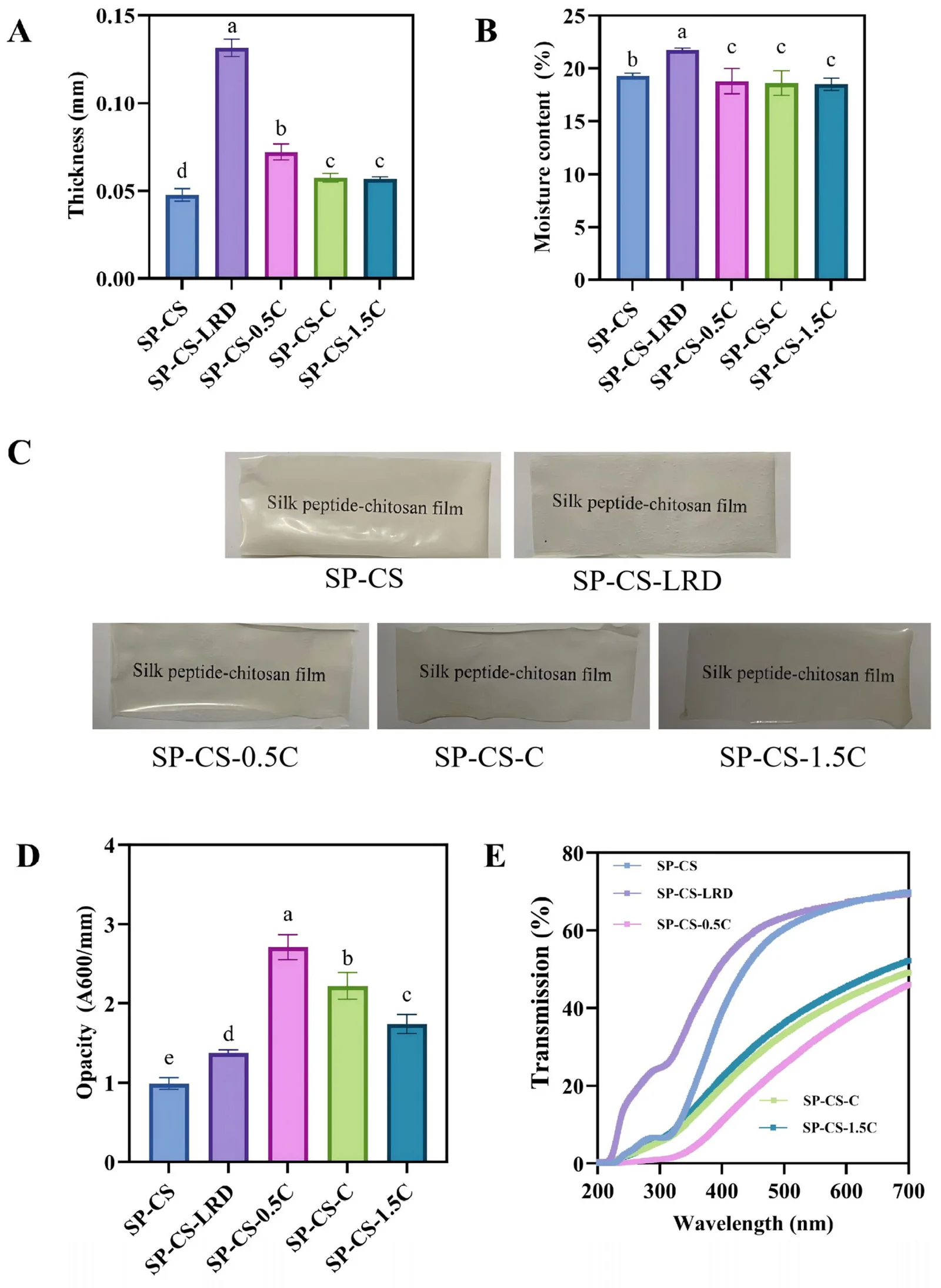
Thickness (**A**), moisture content (**B**), appearance (**C**), opacity (**D**) and transmission (**E**) of the SP–CS films containing different LRD/LAE complexes. Values with different lowercase letters in the same subfigure are significantly different at *p* < 0.05.

The thickness and moisture content of the prepared films are shown in Figure 6A and Figure 6B, respectively. Compared with that of the control SP−CS film, the thickness of the SP−CS−LRD film significantly increased from 0.048 mm to 0.131 mm (*p* < 0.05). The structural compactness of its components generally influences film thickness during the drying process. The inherent tendency of LRD to aggregate likely caused phase separation between LRD and the SP−CS matrix, thereby disrupting the structural integrity of the film and resulting in increased thickness [42]. Conversely, the thickness of the SP−CS films containing LRD/LAE complexes gradually decreased. Notably, the SP−CS−1.5C film had the lowest thickness (0.057 mm), which could be because the LRD/LAE complexes were better dispersed in the SP−CS at this concentration, resulting in a dense network. In addition, the moisture content of the SP−CS−LRD film increased from 19.28% to 21.72%, which may have been due to the hydrophilicity of the LRD. However, the moisture content of the SP−CS film containing LRD/LAE complexes decreased and approached that of the SP−CS film.

The appearance, opacity and light transmission of the films are shown in Figure 6C, Figure 6D and Figure 6E, respectively. In most cases, the film transparency is determined by the type of biopolymer, the thickness of the film, and the additive distribution [33]. The SP−CS film was a yellowish transparent film. The opacities, however, increased when LRD and LRD/LAE complexes were added. This high transmissibility of the SP−CS−LRD film is attributable to LRD aggregation; it has been reported in previous research that the lack of compatibility between the constituents decreases the transparency because of light reflection or scattering at the interfaces [43]. In the case of the films with LRD/LAE complexes, the opacities were significantly greater than those of the SP−CS and SP−CS−LRD films (*p* < 0.05). The progressive increase is also probably caused by the complexes causing the crystallization of SP (formation of β–sheets), forming crystalline regions that reflect light. Moreover, the films in the complex were thinner, but their calculated opacity values were greater. This is mathematically explained by the dependence of the inversely correlated relationship between the thickness and opacity and the construction of a more light−scattering structure of higher density. The difference in the complexes in terms of the films is related to the variation in the content of the β−sheets and the level of dispersion of the complexes. In agreement with the findings of the opacities, the light transmission of the SP−CS films with LRD/LAE complexes was lower than that of the SP−CS and SP−CS−LRD films (*p* < 0.05), with SP−CS−0.5C recording the lowest light transmission. Ultraviolet (UV) light is known to cause the formation of free radicals, lipid oxidation, the degradation of food nutrients, and the discoloration of foods [44]. Thus, films with good UV barrier properties play a critical role in food preservation. Every SP–CS film exhibited good UV−blocking at 200–300 nm. This is due to the presence of aromatic amino acids in SP, including tyrosine, phenylalanine and tryptophan, which are good UV radiation absorbers at wavelengths below 300 nm [45].

### 3.6. Water Vapor Permeability (WVP)

**Figure 7:**
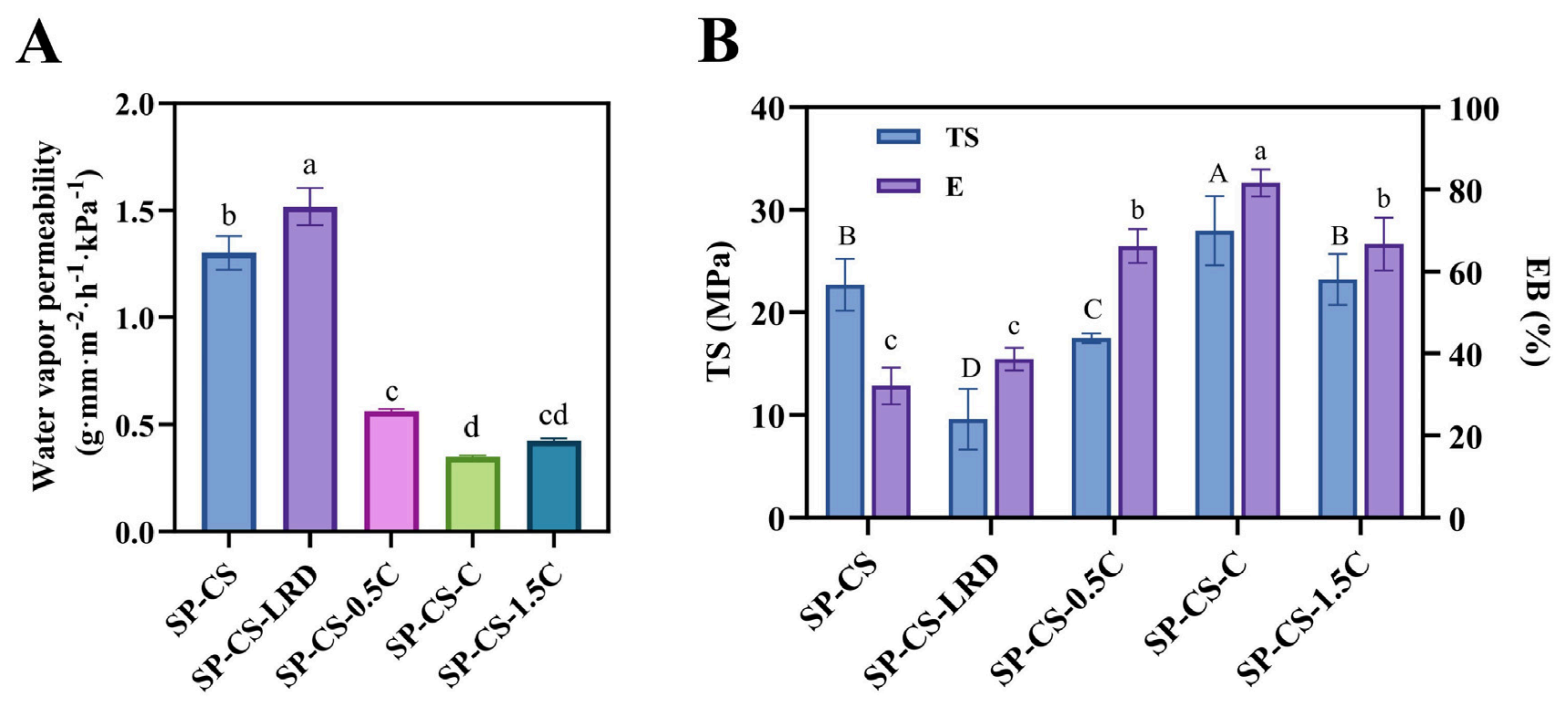
WVP (**A**) and mechanical properties (**B**) of SP−CS films containing different LRD/LAE complexes. In panel (**A**), different lowercase letters denote significant differences (Duncan’s multiple range test, *p* < 0.05). In panel (**B**), capital letters (A–D) indicate significant differences in tensile strength (TS), and lowercase letters (a–d) indicate significant differences in elongation at break (EB) (Duncan’s multiple range test, *p* < 0.05).

Water vapor permeability (WVP) is a metric used to determine how readily a film releases and absorbs moisture from its environment. High WVP allows oil and oxygen to pass through packaging and may accelerate food spoilage and shorten shelf life [46]; as such, it is important to choose packaging materials that have low WVP. As shown in Figure 7A, the WVP of the SP–CS–LRD film increased significantly compared with that of the control by 1.30 to 1.52 g·mm·m^−2^·h^−1^·kPa^−1^ (*p* < 0.05). This increase is attributable to the direct addition of LRD, which is likely to aggregate. This aggregation causes an uneven distribution of the SP–CS matrix, which reduces the uniformity of the film and provides favorable routes through which water vapor can be transported. In contrast, the addition of LRD/LAE complexes significantly reduced the WVP of the SP−CS films (*p* < 0.05). This enhancement is based on the uniform distribution and orientation of the nanosheets [47]. In particular, LAE promotes the exfoliation and dispersion of LRD layers in the matrix, hence forming a tortuous path that effectively increases the diffusion distance of the water molecules. Moreover, the LRD/LAE complexes facilitated the establishment of additional β−sheet structures in the SP, which led to the creation of a tightly packed network of films, further suppressing the flow of water vapor. The SP−CS−C film had the lowest WVP (0.35 g·mm·m^−2^·h^−1^·kPa^−1^). This is likely due to the optimal dispersion of the 2−1 LRD/LAE complex and the establishment of the densest structural network. On the basis of the comprehensive evaluation of mechanical properties (tensile strength: 27.99 MPa; elongation at break: 81.62%), barrier performance (lowest WVP), thermal stability, and banana preservation outcomes, the SP−CS−C formulation (containing 2% LRD and 1% LAE) was identified as the optimal concentration and was therefore selected for the banana preservation assay.

### 3.7. Mechanical Properties

The mechanical properties of the different SP−CS films are shown in Figure 7B. Compared with the neat SP−CS film (TS 22.69 MPa, EB 32.09%), the SP−CS−LRD film showed a marked decrease in tensile strength (TS 9.6 MPa) but an increase in elongation at break (EB 38.62%). The decrease in strength can be explained by the concentration points of stress in the matrix caused by the aggregation and unsatisfactory dispersion of LRD, which disturbed the structural continuity of the film [39]. When LRD was incorporated as an LRD/LAE complex, the exfoliation of the clay layers and their more efficient and uniform dispersion in the SP−CS matrix led to improved mechanical performance. Accordingly, compared with the SP−CS−LRD film, the SP−CS−0.5C film exhibited higher TS and EB, and the higher β−sheet content induced by the 2−0.5 LRD/LAE complex further strengthened the film structure. Among all the formulations, the SP−CS−C film had the highest tensile strength (27.99 MPa) and elongation at break (81.62%), which is attributed to the superior uniformity of the dispersion of the 2−1 LRD/LAE complex, promoting efficient stress transfer at the interface and a uniform stress distribution [39]. Additionally, the SP−CS−C film had the greatest amount of β−sheets, conferring a strong yet flexible network that enables the film to resist external forces. Nevertheless, the mechanical characteristics of the SP−CS−1.5C film were somewhat reduced, which may be due to two factors: first, the lower actual content of LRD in the 2–1.5 LRD/LAE complex (given the same total addition amount), and second, the significantly lower β−sheet content in SP−CS−1.5C than in SP−CS−C, which weakened the structural reinforcement.

### 3.8. Application of the SP−CS Film−Forming Solution Containing LRD/LAE Complexes in Banana Preservation

#### 3.8.1. Appearance

**Figure 8:**
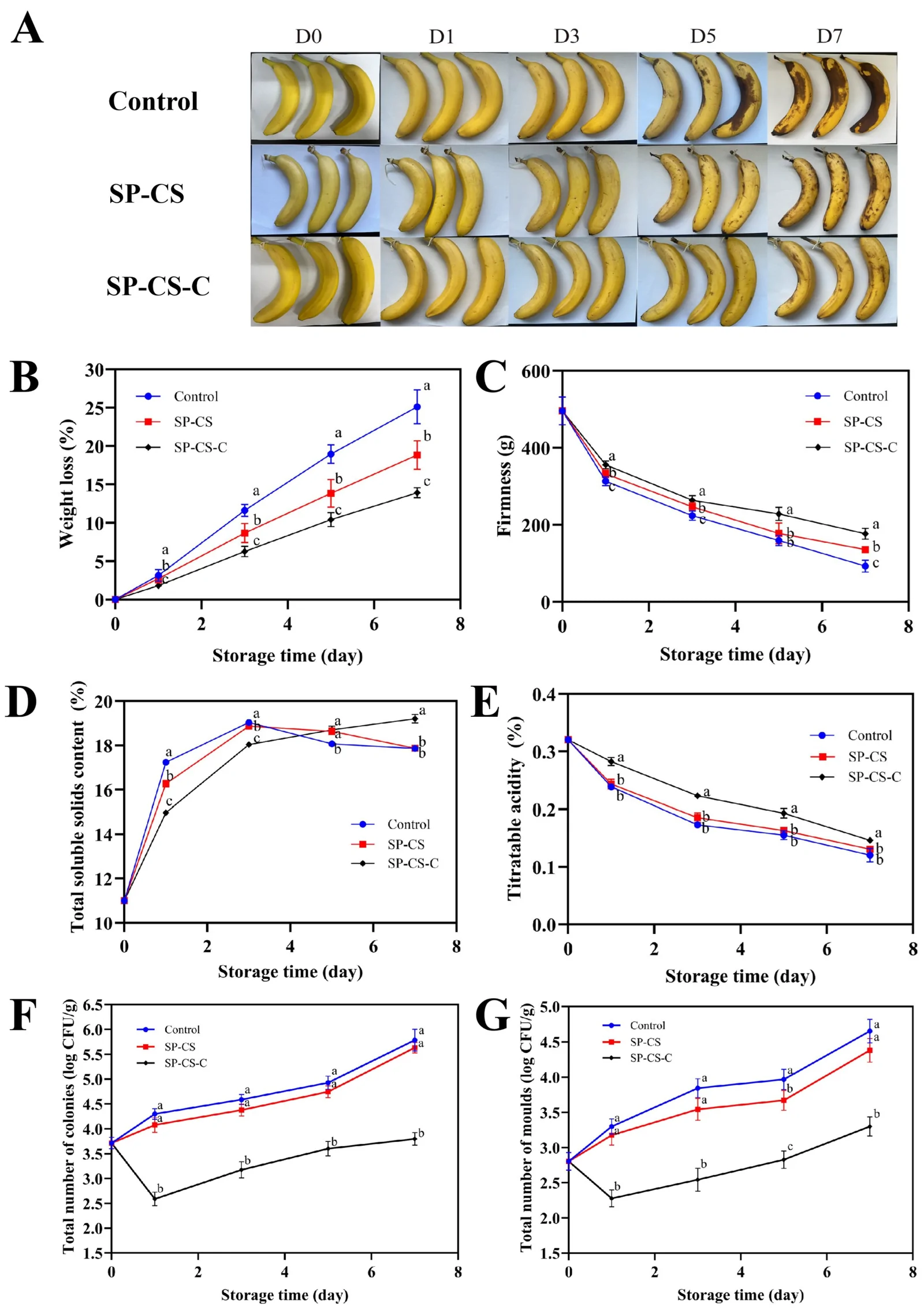
Appearance (**A**), weight loss (**B**), firmness (**C**), TSS (**D**), titratable acidity (**E**) and total number of colonies (**F**) and molds (**G**) of bananas during storage. Different lowercase letters within the same storage time indicate significant differences among treatments (Duncan’s multiple range test, *p* < 0.05).

The appearance of bananas stored in various packages at 25 °C and 50% RH is shown in Figure 8A. Initially, all bananas displayed a vibrant yellow hue. By the fifth day of storage, the bananas in the control group exhibited significant browning along with small black spots. In contrast, those coated with SP−CS experienced only minimal browning and a few black spots, whereas bananas treated with SP−CS−C did not develop significant black spots. The browning was attributed to the oxidation of phenolic compounds in bananas as they ripened, whereas the black spots were caused by anthracnose disease infection during storage [48]. During the storage period, the uncoated bananas experienced extensive decay. Conversely, bananas coated with the SP−CS−C were better than bananas with the SP−CS film and control samples were. This demonstrated that the LRD/LAE complex incorporated into SP−CS films could delay the decay of bananas. The SP−CS coating formed a physical barrier that blocked moisture and oxygen, and the LRD/LAE complex inhibited microbial growth (Figure S1), thus preventing banana browning and disease development and ultimately prolonging the shelf life of the bananas. It should be noted, however, that the casting method used here is suitable for laboratory–scale preparation, and translating it to industrial production poses considerable challenges; the present study therefore provides proof−of−concept evidence, with further research needed before this approach can be applied on a commercial scale.

#### 3.8.2. Weight Loss (WL)

During the postharvest storage of bananas, weight loss occurred because of respiration and moisture evaporation. The weight loss in all the groups gradually increased during the storage period, as shown in Figure 8B. The control group exhibited the greatest weight loss, reaching 25.10% by the end of the storage period. Conversely, compared with the control group, the SP−CS group markedly decreased weight loss to 18.83%. This decrease might be attributed to the water barrier properties of the SP−CS film, which were used to reduce the loss of moisture and, as a result, reduce the loss of water in bananas. The lower water vapor permeability (WVP) and high antimicrobial properties of the SP−CS−C film (reduced water loss and respiratory rate) may explain why the weight loss rate of the SP−CS−C was the lowest (13.92%). Weight loss revealed that compared with the uncoated and the SP−CS film, the SP−CS−C film was likely to reduce the weight loss of the bananas more.

#### 3.8.3. Firmness

The drying of banana fruits, breakdown of starch, depolymerization of matrix polysaccharides and pectin, and loss of neutral sugars in pectin side chains may all play a role in the process of hardness in bananas. Additionally, cell wall–degrading enzymes such as pectin methyl esterase, polygalacturonase, pectin lyase and cellulase were also used to determine the hardness of the fruit [24]. Thus, firmness is among the most important markers for determining the quality of stored bananas. As shown in Figure 8C, the firmness of the bananas in both groups tended to decrease with increasing storage time. The hardness of the control group decreased rapidly from 495.66 g to 92.55 g after storage. The SP−CS and SP−CS−C samples were stronger than the control group, which was 135.79 g and 176.94 g, respectively. Compared with the other two materials, the SP−CS−C coating package could be successful at regulating the reduction in banana hardness (*p* < 0.05). This could be because the SP−CS−C coating prevented the growth of microbes, hence lowering the utilization of nutrients by the microbes in the bananas. Moreover, the barrier characteristics of the SP−CS−C were lower, which may influence moisture and gas exchange and decrease enzyme reactions and respiration rates, thereby slowing the softening of banana fruits.

#### 3.8.4. Titratable Acidity (TA) and Total Soluble Solids Content (TSS)

Organic acids such as malic acid, oxalic acid and citric acid are also common in fruits, and they not only add flavor but are also used to determine the freshness of fruits. It has been established that the presence of organic acids in bananas might be neutralized or esterified during storage, leading to the gradual depletion of organic acid contents [49]. The titratable acidity (TA) content in bananas, which is shown in Figure 8D, decreased over time of storage. Initially, the TA content was 0.32%. The TA contents in the SP−CS and SP−CS−C groups after one week of storage were 0.13 and 0.15, respectively, which were higher than that in the control group (0.12). These findings indicated that the SP−CS−C coating has the potential to reduce the rate at which TA decreases.

Changes in overall soluble solids (TSSs) are related to the ripening of fruits and vegetables [50]. The oxidation and degradation of nutrients in bananas (starch, lipids, etc.) resulted in the development of soluble polysaccharides, which subsequently resulted in an increase in TSS [51]. Figure 8E shows that the TSS of the control and SP−CS groups started to increase but then decreased during the storage period, and the TSS of the SP−CS−C group increased steadily during the storage period. The starch in bananas was converted to glucose, and this helped to increase the TSS to high levels, which reached their optimal point when the bananas were fully ripened. The subsequent respiration of bananas requires the consumption of nutrients. When the consumption rate outpaced the conversion of total soluble solids, the TSS of bananas began to decline. Bananas in the control and SP−CS groups reached full ripeness on day 3, reaching the peak of the TSS and then declining. In contrast, the SP−CS−C group demonstrated a general increase in TSS, indicating that this coating could effectively delay the ripening of bananas and inhibit the increase in TSS.

#### 3.8.5. Antibacterial Activity

As shown in Figure 8F and Figure 8G, the total number of colonies and molds on bananas in the control group noticeably increased during storage, reaching 5.78 and 4.65 log CFU/g, respectively, at the termination of the storage period. The SP−CS−coated bananas did not show obvious advantages, indicating that the SP−CS coating had little antimicrobial effect. The antimicrobial activity of the SP−CS−C group was notable, with the number of colonies and molds reaching 3.80 and 3.30 log CFU/g, respectively. The antimicrobial effect could be attributed to the presence of LAE in the SP−CS−C coating, which possessed broad−spectrum antimicrobial properties [52]. The antibacterial activity of the coating film formed from the SP−CS−C film−forming solution against *E. coli* and *S. aureus* is shown in Figure S1. This characteristic effectively prevents fruit spoilage during storage, such as during the growth of the banana pathogen anthrax, thus prolonging the shelf life of bananas.

## 4. Conclusions

In this study, a solution−cast chitosan/silk peptide (SP−CS) film incorporating a lysozyme−derived antimicrobial peptide/diacetate ester (LRD/LAE) complex was developed, with particular attention given to how LAE−modified LRD influences the structure and performance of the composite films. The LRD/LAE complex improved the dispersibility of LRD within the SP−CS matrix and was associated with enhanced SP–CS compatibility, resulting in improved mechanical, thermal, and antibacterial properties, which could be attributed to modified amide spectral characteristics and crystalline arrangement within the SP−CS network. When applied as a coating, the SP−CS−C film−forming solution reduced weight loss in bananas while better maintaining the firmness, titratable acidity, and soluble solid content during storage. Nevertheless, the migration behavior and release kinetics of LAE were not examined, and comprehensive safety and biodegradability assessments remain to be carried out. Future work should therefore first clarify the migration and release kinetics of LAE from the complex within the film matrix and then evaluate the biodegradability of the films under different environmental conditions. Because the present design compared only the control, SP−CS, and SP−CS−C films, the individual contributions of LRD and LAE cannot yet be fully separated; a full factorial study covering SP−CS, SP−CS−LRD, SP−CS−LAE, and SP−CS−LRD/LAE films at varying ratios is accordingly planned to distinguish synergistic effects from additive effects. The scalability of the casting method for industrial production—including roll−to−roll alternatives—should also be examined, along with the efficacy of the coating on a wider range of perishable produce and the development of predictive shelf−life models based on observed changes in quality attributes. Together, these efforts will be important for evaluating the potential of the SP−CS film containing the LRD/LAE complex in food preservation.

## Figures and Tables

**Table 1:** Sample codes and compositions (represented as portions by weight).

Sample Code	SP (g)	CS (g)	2% Acetic Acid (mL)	LRD/LAE Complex (g)
SP–CS	2.4	1.6	200	/
SP–CS–LRD	2.4	1.6	200	0.2
SP–CS–0.5C	2.4	1.6	200	0.2
SP–CS–C	2.4	1.6	200	0.2
SP–CS–1.5C	2.4	1.6	200	0.2

## Data Availability

The original contributions presented in the study are included in the article/Supplementary Material, further inquiries can be directed to the corresponding authors.

## Supplementary Materials

The following supporting information can be downloaded at https://www.mdpi.com/article/10.3390/foods15213821/s1, Figure S1. Antimicrobial diameter and antimicrobial growth curve of SP-CS films against *E. coli* and *S. aureus*.
