# Supplementary material for: Laponite®/Ethyl Lauroyl Arginate Complex–Functionalized Silk Peptide/Chitosan Edible Coating for Banana Preservation
Source: Foods. 2026 Oct 26;15(21):3821. doi: 10.3390/foods15213821 (PMC13763009; doi:10.3390/foods15213821)
Supplement: Supplementary file 1 [file foods-15-03821-s001.zip › foods-4548304-supplementary.pdf]

Article

Laponite®/Ethyl Lauroyl Arginate Complex–Functionalized Silk Peptide/Chitosan Edible Coating for Banana Preservation

Congcong Zhao [1,2], Dongxia Hu [1], Nina Mo [1], Guanghui Wang [1], Hongying Lin [2], Minmin Tang [3], Yayuan Zhang [4], Xiaozhi Tang [1], Ru Zhao [2,*], Tingli Liu [2] and Zhenjiong Wang [2,*]

1 College of Food Science and Engineering, Nanjing University of Finance and Economics, Collaborative Innovation Center for Modern Grain Circulation and Safety, Nanjing 210023, China
2 School of Food Science, Nanjing Xiaozhuang University, Nanjing 211171, China
3 Key Laboratory of Food Contact Materials Safety, State Administration for Market Regulation, Jiangsu Product Quality Testing & Inspection Institute, Nanjing 210007, China
4 Institute of Agro–Products Processing Science and Technology, Guangxi Academy of Agricultural Science, Nanning 530007, China
* Correspondence: 2021044@njxzc.edu.cn (R.Z.); wangzhenjiong@gmail.com (Z.W.)

2. Materials and methods

Antibacterial properties of the coating film

The antibacterial properties of the coating film against *E. coli* and *S. aureus* were evaluated using both the disk diffusion and liquid medium methods (Xu, et al., 2020). A single colony of *E. coli* and *S. aureus* was inoculated into 5 mL LB broth and incubated at 37°C for 24 h. The inoculums were then diluted and adjusted to 10^8 CFU/mL for further experimentation. The cooled and solidified NA Agar dish was coated with 120 μL of bacterial suspension of *E. coli* and *S. aureus* (10^5 CFU/mL), respectively. The film was cut into a 6mm diameter disc and placed on the NA Agar dish containing the bacterial solution. Then the petri dish was placed in a refrigerator at 4°C and spread for 24 h. It was then cultured in an incubator at 37°C for 24 h. Finally, the diameter of the inhibition zone was ultimately measured at least in triplicate. The 50 mg film was immersed in a tube with 10 mL of LB broth for 1 h. Subsequently, 20 μL of bacterial suspensions containing E. coli and S. aureus (10^8 CFU/mL) were introduced into the LB broth, respectively. The mixtures were incubated at 150 rpm at 37°C, with the OD600 values measured at predetermined intervals using a plate reader (SpectraMax 190, Molecular Devices, Shanghai, China) to generate microbial growth curves. LB broth acted as the reference, while the bacterial suspensions served as blank control.

3. Results and discussion

Antibacterial activity of the coating film formed from the SP-CS-C film-forming solution

The inhibitory zones of the Fig.S1 also illustrated the antibacterial activity of the coating film formed from the SP-CS-C film-forming solution against *E. coli* and *S. aureus*

(Fig.S1A and S1B）. The SP-CS and SP-CS-LRD did not exhibit antibacterial properties. However, the addition of the complex led to an increase in the diameter of the inhibitory zones with the increasing concentration of LAE in the complex (p<0.05). The SP-CS-0.5C film exhibited no inhibitory effect against *E. coli* but significantly inhibited *S. aureus* with a zone of 28.33 mm. The inhibitory zones for *E. coli* and *S. aureus* of the SP-CS-1.5C film were 24.00 mm and 36.00 mm, respectively. The antibacterial activity of the SP-CS films containing LRD/LAE complexes mainly stems from the LAE, with a markedly higher inhibitory effect on *S. aureus* compared to *E. coli*. This difference was related to the presence of lipopolysaccharides on the outer membrane of Gram-negative bacteria, which may impede LAE from reaching its target sites in

the cell membrane, rendering Gram-negative bacteria more resistant to LAE (Li, et al., 2021). The growth curves of *E. coli* and *S. aureus* by all the films were shown in Fig. S1C, D. Bacteria in all groups grew rapidly in the first 8 hours, followed by entering a stable growth phase. Compared to the bacterial control group, the SP-CS group showed a relatively slower growth curve but eventually approached that of the bacterial control group. Meanwhile, the growth curves of *E. coli* and *S. aureus* by SP-CS-LRD basically overlapped with the bacterial control group. This indicated that SP-CS and SP-CS-LRD film had no significant long-lasting antibacterial effect. The antibacterial activity of SP-CS and SP-CS-LRD was primarily attributed to CS. The CS in SP-CS-LRD may bind with aggregate LRD, potentially leading to a less effective antibacterial activity compared to the SP-CS. The growth curves and OD value of *E. coli* and *S. aureus* by SP-CS films containing LRD/LAE complexes were significantly lower than those of the bacterial control group, SP-CS and SP-CS-LRD groups, suggesting that the complex could significantly inhibit bacterial growth, thereby enhancing the antibacterial activity of the film.

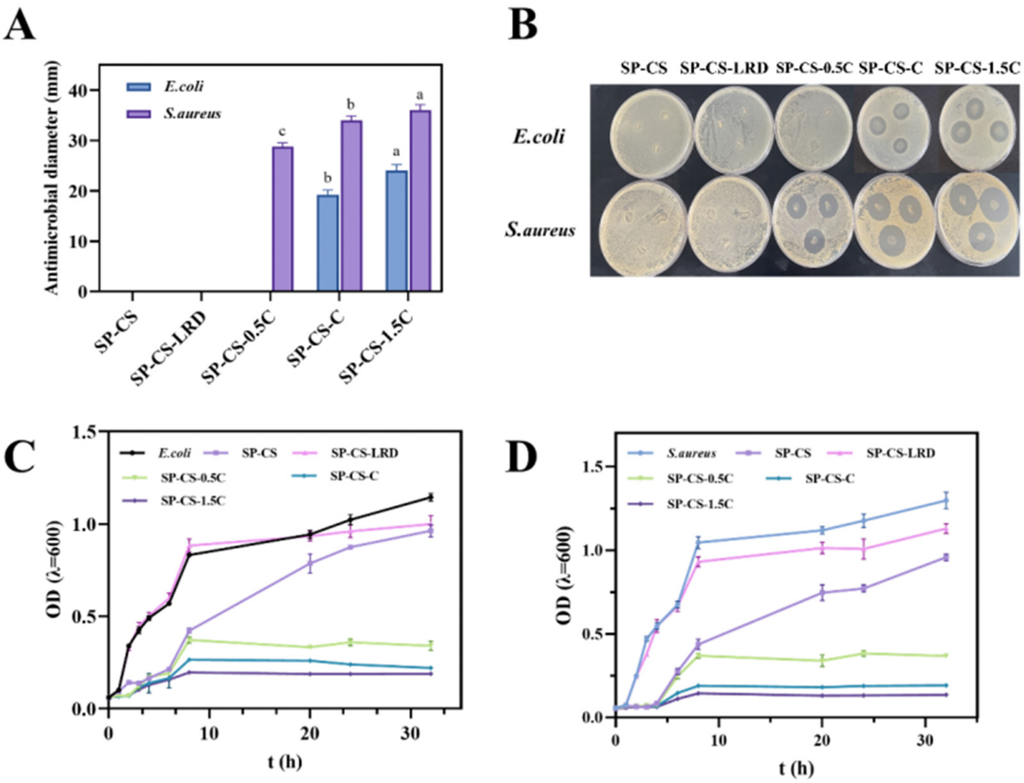

Figure S1. Antimicrobial diameter and antimicrobial growth curve of SP-CS films against *E. coli* and *S. aureus*.
